# The evolving role of pharmacists in depression care: a scoping review

**DOI:** 10.1007/s11096-024-01759-1

**Published:** 2024-07-15

**Authors:** Ala’ Shalash, Monica Zolezzi

**Affiliations:** 1 Clinical Pharmacy Department, Lehbi Renal Care, Riyadh, Kingdom of Saudi Arabia; 2 Clinical Pharmacy and Practice, College of Pharmacy, QU Health, Qatar University, Doha, Qatar

**Keywords:** Depression, Management, Pharmacist, Pharmaceutical care, Pharmacy services, Pharmacy

## Abstract

**Background:**

Worldwide, depression is known to contribute significantly to the global burden of disease. Considering pharmacists are among the most approachable healthcare providers, they are well-placed to assist people with depression achieve positive treatment outcomes.

**Aim:**

The primary aim was to examine the evidence regarding pharmacists’ roles in interventions, outcomes, and barriers to implementation within depression care globally, with the secondary aim focusing on the Arab region.

**Method:**

A scoping review was conducted according to the PRISMA-ScR extension guidelines and the Joanna Briggs Institute framework, using Scopus, Cochrane, ProQuest, and Medline databases for studies worldwide and within the Arab region (22 Arab-league countries). Article selection, along with data extraction, analysis, and narrative synthesis were performed independently by two reviewers. Discrepancies were resolved by consensus.

**Results:**

Forty studies reporting various roles and services provided by pharmacists in depression management were included. Most articles (24) described studies on pharmacist-led specific/single interventions/management strategies, and 16 described studies in which pharmacists provided comprehensive or team-based services. The majority of studies reported positive impact on patient outcomes. In accordance with the secondary aim, only three studies assessed various pharmacists’ services for people with depression in the Arab region. Barriers to effective depression-related care included time constraints and training needs.

**Conclusion:**

This scoping review supports the expanding role of pharmacists in depression management. The interventions, impact, challenges, and research gaps identified serve as preliminary evidence for advocating for an expanded pharmacists’ scope of practice in mental health, both globally and in the Arab region.

**Supplementary Information:**

The online version contains supplementary material available at 10.1007/s11096-024-01759-1.

## Impact statements

- Pharmacists contribute to depression management by providing their services in various ways and settings. However, a standardized definition of the role of pharmacists in depression management is lacking.
- There is a significant gap in the provision of care to people with depression, with their role found to be restricted to prescription dispensation, the provision of product information, and counseling on the management of antidepressant side effects. This gap is more significant in the Arab region compared to the global landscape.
- Despite growing interest in mental health, there are several challenges both globally and in the Arab region, mostly in regard to the limited scope of pharmacy practice that limit pharmacists from providing comprehensive care to people with depression.

## Introduction

Depression is a serious global public health concern with a high prevalence, recurrence, and mortality rate [1]. The World Health Organization (WHO) reports that more than 280 million people worldwide experience depression [2]. The recent COVID-19 pandemic has been associated with a sharp rise in the number of depressive disorders worldwide [3]. A large body of literature indicates that depression places a significant burden on society, both clinical and economic (e.g., direct costs that include resources spent on inpatient and outpatient care and therapies, suicide-related costs, and loss in productivity) [1–5].

The use of effective treatments, both pharmacological and psychotherapy, offers hope for people with depression. However, in a study conducted in 21 countries, results indicated that most people with depression do not receive adequate treatment [6]. This finding equated to one in five people in high-income countries, and one in 27 in low-, or low-to-middle-income countries, highlighting the need to implement fundamental transformations involving community education and outreach, beyond what it is currently being offered in primary and secondary care.

Over time, the role of the pharmacist has expanded to encompass patient-centered clinical services for nearly every type of illness, including mental health disorders. As pharmacists are among the most accessible primary healthcare professionals, they can provide numerous services for people with mental health conditions, such as screening and referral, education, medication counseling, monitoring therapy and supporting treatment adherence [7–9]. In a recent white paper, the American Pharmacists Association urged community pharmacists to be more actively engaged in managing depression in order to improve patient outcomes and quality of life [10]. Similarly, the European Society of Clinical Pharmacy Special Interest Group on Mental Health advocated for enhanced and standardized involvement of clinical pharmacists in depression management [11].

Despite the above reports, there is still a significant gap in the scope of practice among pharmacists when providing care to people with depression. Their function has been reported to be restricted to dispensing medications, the provision of product information, and counseling on the management of antidepressant side effects [12, 13]. In the Arab region, which is defined as and comprising of 22 nations within the Arab league, this gap is even more significant, with very limited studies reporting on the role of pharmacists in depression care [14, 15]. Data from the region reports almost 12 million disability-adjusted life years due to mental illness, with depression and anxiety being the most common disorders [16]. Given the region’s unique cultural, social, and healthcare landscape, a review of such literature can aid in understanding the challenges and opportunities of pharmacists in depression care in countries within the Arab world. This scoping review could serve as a valuable resource for organizations and healthcare professionals through offering insight into the pharmacists’ current practice reported in relation to depression care, addressing systemic barriers, and guiding the development of future evidence-based interventions.

### Aim

The aim of this scoping review was twofold. It primarily aimed to comprehensively map the literature discussing the role of pharmacists in depression care worldwide, focusing particularly on interventions and management strategies, outcomes assessed, and barriers to effective depression care. As a secondary aim, it also aimed to explore the current landscape of pharmacist-led interventions specifically within the Arab region, comprising the 22 countries belonging to the Arab league (Bahrain, Comoros, Djibouti, Egypt, Iraq, Jordan, Kuwait, Lebanon, Libya, Mauritania, Morocco, Oman, Palestine, Qatar, Saudi Arabia, Somalia, Sudan, Syria, Tunisia, the United Arab Emirates, and Yemen, Algeria). This review will provide a comprehensive understanding of the pharmacist’s role in depression care and guide future initiatives in the field in countries within the Arab region and globally.

## Method

A scoping review was undertaken to examine the characteristics of pharmacists’ roles in the management and care provision of depression in the literature [17, 18]. Moreover, the heterogeneity of the evidence concerning this topic calls for an exploration of the landscape of the literature to help guide future research and systematic reviews.

This review used the Joanna Briggs Institute (JBI) methodology for scoping reviews and other related guidelines [19–21]. This framework was used to provide an expert-developed, structured, comprehensive, and transparent approach to this scoping review. A literature search of studies addressing pharmacists’ roles in depression care globally was conducted in MEDLINE, SCOPUS, Cochrane Library and ProQuest databases. The scoping review included only full-text English publications from database inception until 31 of December 2022. To determine if there were studies specifically conducted in the Arab region, the following search terms were used: “Arab countries” or “Arab” or “Gulf cooperation countries” or “GCC” or searching by the name of each Arab country. The search was undertaken in English, as English is the predominant language of scientific communication and publication within the Arab region.

### Search strategy

Keywords and medical subject headings (MeSH) terms used included: “pharmacist”, “depression”, “depressive disorders”, “pharmacy services”, “management”, and “pharmaceutical care”. To expand the search and find all pertinent publications, a combination of search terms, Boolean operators (OR, AND), and truncations (*) were employed as necessary. Appendix 1 includes specific search strings and filters for each database.

### Article selection

One investigator (AS) conducted the database search. The titles of studies identified through the databases were imported into Al Rayyan® software which was used to identify and delete duplicates. Title and abstract screening were independently performed by two reviewers (AS and MZ) in accordance with predetermined eligibility criteria (Table 1). Full text screening for the included studies was similarly performed. In case of discrepancies, consensus was reached through revisiting the eligibility criteria and discussion between the two reviewers, or consulting with a third external reviewer. Furthermore, a supplementary manual search was conducted on all the reference lists and bibliographies of included studies, including those of systematic reviews (SRs) and narrative reviews (NRs), to ensure that no relevant studies were overlooked. Potentially relevant studies were examined individually to see if they met the inclusion criteria for our scoping review.

**Table 1:** Eligibility criteria for the scoping review utilizing ECLIPSE* framework

	Inclusion	Exclusion
Expectation	Pharmacist care improve patient depression overall outcomes	Articles reporting on patient or pharmacists’ perspectives on their potential roles in managing depression
Client group (ECLIPSE)	Patients with depressive disorder ± other health conditions	If the main service was for the comorbid condition and not MDD-
Location, Service (ECLIPSE)	Community/hospital/clinic settings where pharmacy services are provided	–
Impact (ECLIPSE)	Pharmacist Interventions (Depression Screening, Depression treatment/education and monitoring, collaborative care and pharmaceutical care), Outcome assessment (positive depression screenings, referrals to GPs and/or other healthcare providers, depression symptoms and severity, and medication adherence), Identified barriers to these interventions	–
Professionals (ECLIPSE)	Pharmacists (community/clinical), multidisciplinary team if pharmacist was part of the team	Multidisciplinary but pharmacists not part of the team
Evaluation (ECLIPSE)	Scoping review	
Language	English	Other languages
Type and year of publication	Full-text peer reviewed papers reporting data from primary research (e.g., RCT), review articles including systematic reviews/scoping reviews/narrative reviews and grey literature	Conference abstracts, and book reviews, were excluded
Accessibility	Abstract and full text fully accessible	No accessibility to the full text or abstract

*ECLIPSE, Research question framework detailing expectation, client group, location, impact, professionals, and evaluation; MDD, Major Depressive Disorder; GP, General Practitioner; RCT, Randomized Control Trial

### Data charting process and synthesis

A data collection form was designed using Microsoft Word® software. To facilitate the data analysis, studies were categorized according to the type of pharmacist interventions/management strategies reported. The following data was extracted, whenever feasible: author(s), year of publication, country, study design, setting, sample size, pharmacist intervention(s), and outcome reported relevant to pharmacist intervention(s). This categorization was informed by an initial screening literature review and was developed to aid examining the evidence in terms of the extent of the pharmacists’ role within the reported interventions. The three categories were as follows:

- Category 1: *‘Pharmacist-led specific/single depression interventions/management strategies’:* Included studies reporting on single interventions or specific strategies implemented and executed by pharmacists to assist patients in the management of depression, including depression screening, referral of patients to general practitioners (GPs) or other mental health services, treatment follow-up, patient education and counseling, and promotion of treatment adherence.
- Category 2: ‘*Pharmacist-led comprehensive depression management strategies*’*:* Included studies reporting on the provision of comprehensive pharmaceutical care services for the management of depression, in which pharmacists were involved in the identification of drug-related problems, and in the development, implementation and monitoring of an individualized pharmaceutical care plan for people experiencing depression.
- *Category* 3: '*Pharmacists’ collaborative care practices in depression management*'*:* Included studies reporting on pharmacist participation in depression management services in which they worked in close collaboration with one or more health care professionals, through a collaborative care agreement and/or shared decision making (SDM).

For these studies, the depression-related outcome of the specific pharmacist-led intervention/management strategy (e.g., improvement in symptoms, decrease in severity, adherence, quality of life, patient satisfaction) and the outcome measure utilized (if reported) were also retrieved. Barriers identified in these studies on the pharmacist’s role in depression care were analyzed separately. As per the JBI methodology guidance for scoping reviews, no quality assessment was deemed necessary to be performed [20–22].

## Results

**Fig. 1:**
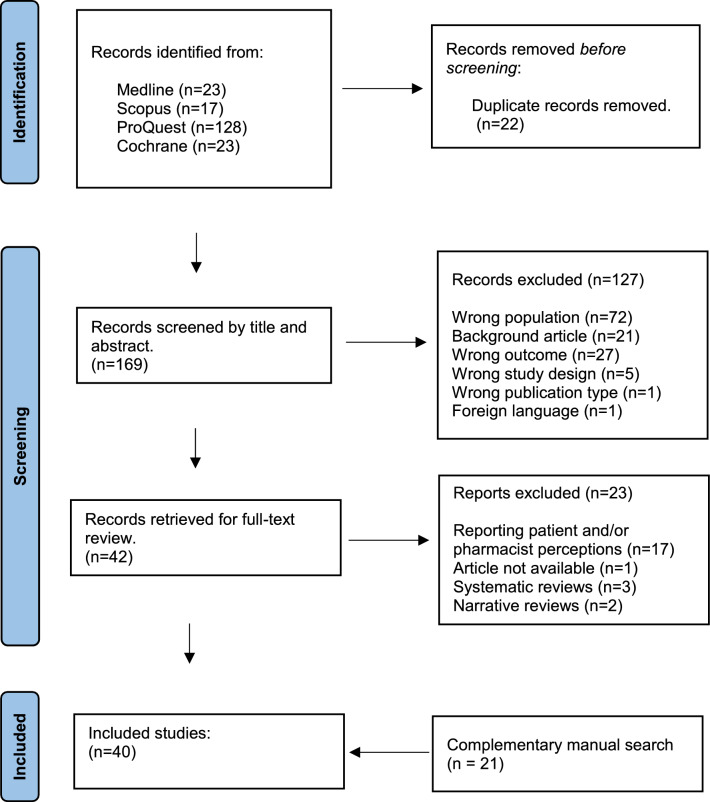
PRISMA diagram reporting the databased used, the number of records screened by title and abstracts, and full-text articles retrieved

As illustrated in Fig. 1, the initial search in the four databases yielded 191 articles. After removing the duplicates (n = 22), 169 records were initially screened by title and abstract, of which 127 were excluded. Of the remainder 42 records that were fully reviewed, only 19 were eligible for inclusion. A total of 21 articles from the complementary manual search were also eligible for inclusion, yielding a total of 40 articles included in this scoping review.

### Characteristics of the studies

Over half of the studies (n = 23/40, 57.5%) were conducted in North America. The other studies originated from the Netherlands (n = 3/40, 7.5%), Spain (n = 2/40, 5%), Australia (n = 2/40, 5%), Thailand (n = 1/40, 2.5%), Bulgaria (n = a 1/40, 2.5%), Japan (n = 1/40, 2.5%), Brazil (n = 1/40, 2.5%), Bosnia and Herzegovina (n = 1/40 = 2.5%), Israel (n = 1/40, 2.5%), and Sweden (n = 1/40 = 2.5%) and only three studies were conducted in Arab countries (KSA, Kuwait and Syria/Jordan).

Half of the studies were conducted in hospitals or affiliated outpatient clinics (n = 20/40, 50%), while the other half were conducted in community pharmacies (n = 19/40, 47.5%) or nursing homes (n = 1/40, 2.5%). The majority of the studies (24/40, 60%) reported on pharmacist-led specific/single depression interventions or management strategies [22–45], four studies (n = 4/40, 10%) reported on pharmacist-led comprehensive pharmaceutical care services for patients with depression [46–49], and 12 studies (n = 12/40, 30%) reported on pharmacists’ collaborative care practices in depression management [50–57].

### Categories of interventions

#### Pharmacist-led depression interventions/services

As summarized in Table 2, two main pharmacist-led depression interventions/services were provided: Among the 24 studies within this category (category 1), 13 studies reported on depression treatment education/monitoring (54.2%, 13 out of 24) [19, 23, 24, 27, 28, 33, 35–41]. and 11 studies reported on depression screening (11/24, 45.8%) [22, 24–26, 29, 30, 33–36, 38]. Outcomes assessed in these studies included positive depression screenings, referrals to GPs and/or other healthcare providers, depression symptoms and severity, and medication adherence. Although various types of screening tools were used, the 9-question Patient Health Questionnaire (PHQ-9) was the most common across all the studies. All the screening interventions positively identified individuals at risk or with depression (between 4–70.7% of individuals screened), and the majority resulted in referrals to GPs or other healthcare providers. Furthermore, six patients out of the 11 positively screened for depression (54.5%) were referred either for further assessment or for starting treatment [22, 26, 30, 33, 35, 38], The most commonly reported outcome for pharmacist-led depression treatment education/monitoring interventions was adherence rate (9/13, 69.2%) [28, 31, 32, 37, 39–42, 45]. Prescription refills, clinic visit frequency, patient self-report, electronic pill containers, and percentage of missed doses were used to assess adherence. The majority of the studies reporting adherence rate as an outcome (7/9, 77.8%) showed improvements depression treatment adherence as a result of the intervention [28, 31, 32, 39, 41, 42, 45]. Depression symptom severity and quality of life were the second most commonly reported outcome for pharmacist-led depression treatment education/monitoring interventions (8/13, 61.5%) [23, 27, 33, 37, 42–45]. The majority of these studies (6/8, 75%) showed the pharmacist intervention resulted in improvement in depressive symptoms, decrease in symptom severity or improved quality of life [23, 27, 31, 37, 44, 45]. Other outcomes reported in the studies under this category included depression knowledge, attitudes, and beliefs (KAB) and patient satisfaction with the services [28, 35, 36, 39]. On the other hand, some studies reported no significant differences on patient-related outcomes [37, 40, 42, 56].

**Table 2:** Characteristics of studies evaluating pharmacist-led specific/single depression interventions/management strategies

References	Study design	Country	Setting, sample size	Interventions/management strategies	Reported results/outcome measures
Ballou [22]	Single center, single arm, uncontrolled study	USA	Setting: community pharmacy Sample size: (n = 77)	Screening	Positive screening n = 18 (23%) Screening Scale used: PHQ-9 Referral for additional treatment n = 18 (23%)
Alkoudsi [23]	RCT	Jordan/Syria	Setting: Community pharmacies Sample size: (n = 118) from both Syria (n = 60) and Jordan (n = 58)	Education and monitoring	Main outcome measured: Improvement in depressive symptoms Scales used: BDI The intervention group showed substantial reductions in their mean BDI scores compared to the control group (Syria: 26.5 ± 12.6 vs. 22.9 ± 12.2, * p* < 0.001; Jordan: 17.7 ± 11.0 vs. 15.8 ± 11.1, * p* < 0.049)
Wilso et al. [24]	Prospective cohort study. Single-center, single arm, uncontrolled	USA	Setting: Community pharmacy Sample size: (n = 57)	Screening	Positive screening n = 11 (19.3%) Screening scales used: PHQ-9, BDI-II, and GDS PHQ-9 was the most widely and preferred scale tool utilized Response rate from PCPs who had been notified of positive screening n = 3 (27%) None initiated treatment based on the screening results
Kondova [25]	Single center, Single arm uncontrolled study	Bulgaria	Setting: Community pharmacy Sample size: (n = 83)	Screening	Positive screening n = 58 (70%) Screening scales used: PHQ-2, if positive, PHQ-9 was used to assess depression severity Depression severity assessment: n = 1 mild depression, n = 16 moderate depression, n = 5 moderately severe depression No interventions were reported following screening
O’Reilly [26]	Interventional cohort study Multi-center, single arm, uncontrolled	Australia	Setting: Community pharmacy Sample size: (n = 41)	Screening	Positive screening n = 29 (70.7%) Number referred to other HCP n = 29 (70.7%) Screening scales used: PHQ-9, WHO-5 and Beyond Blue Depression Checklist PHQ-9 was the most widely used tool (n = 22) Referred to GP n = 25 (86.2%), psychologist n = 3 (10.3%), unknown n = 1 (3.3%)
Phimarn [27]	A two-phase, cross-sectional study, group vs individual counseling	Thailand	Setting: Community pharmacy Sample size: (n = 68)	Education and monitoring	Main outcomes measured: Depression severity and QOL by standard CES-D and SF-36 scales, respectively Symptom severity score as lower in the group who received individual counselling from a trained pharmacist than in the group receiving group counselling (17.7 ± 4.5 vs 20.1 ± 4.6, * p* = 0.038) At week 16, counseling increased the QOL mean score of physical health significantly (7.8 points for group counseling and 6.7 points for individual counseling), but only individual counseling significantly increased the mean score of mental health, going from 39.9 ± 9.9 to 43.1 ± 8.4 points
Klang [28]	Exploratory prospective, non-randomized, open-label, naturalistic observational study	Israel	Setting: Community pharmacy Sample size: (n = 4252)	Education and monitoring	Main outcome measured: Adherence rate Adherence method used: Pharmacy records refills At 1 month and at 6 months, the adherence rate in the intervention group was higher than in the TAU group (71% vs. 57% and 55% vs. 15.2%, respectively)
Tingen [29]	Interventional cohort study	USA	Settings: Outpatient clinics by clinical pharmacist Sample size: (n = 15)	Screening	Positive screening n = 2/15 (13.3%) Screening scales used: PHQ-2 Eight of the 15 patients (53%) had face-to-face interviews, and 7 had phone interviews (46%) All who screened positive were informed about clinic services (availability of social workers) and advised to follow up with their GP for further evaluation (number who followed up on any of these recommendations were not reported)
Rosser [30]	Prospective cohort study Multi-center, single arm, uncontrolled	USA	Setting: Community pharmacy Sample size: (n = 3,726)	Screening	Positive screening n = 17 (25.3%) Referred n = 17 (100%) Screening scales used: PHQ-9 For those who were referred treatment (pharmacological or psychotherapy) was initiated in n = 6 (35.3%) and modified in n = 4 (23.5%) patients, n = 5 (29.4%) did not receive treatment initiation or modification, n = 2 (11.8%) were lost to follow-up
Rubio-Valera [31]	A six-months follow-up naturalistic parallel-group multicenter RCT	Spain	Setting: Community pharmacies Sample size = 179 patients	Education and monitoring	Main outcome measured: Adherence rate, KAB and QoL Scales used: PHQ-9 to assess the clinical severity of depression, Euroqol-5D scale to assess HRQOL, and patient-satisfaction questionnaire Intervention group more likely to remain adherent both at 3 months (67.7% vs 83.3%) and at 6-months (46.3%vs.67.3%), * p* = 0.20 NNT was 5 (5 patients need to have intervention in order to avoid one non-adherent patient) The intervention group showed statistically greater overall improvement in HRQOL than the control group (0.25 vs. 0.14), * p* < 0.001
Rubio-Valera [32]	RCT Cost effectiveness study	Spain	Setting: Primary Care Health Centers Sample size: 30 GP	Education and monitoring	Main outcomes measured: Adherence to antidepressants, clinical symptoms, QALYs Pharmacist intervention to IG showed a probability of being cost-effective in adherence and QALYs with a 71% chance of improving medication adherence and a 75% chance of improving the patients' quality of life
Ito [33]	Single center, retrospective cohort study	Japan	Setting: Outpatient oncology clinic Sample size: (n = 520)	Screening	Positive screening n = 26 (5%) Screening scale used: DIT Referred to psychiatrists n = 26 (5%) If a DIT-positive patient declined referral to mental health services, the pharmacist educated the patient on depression management No interventions were reported following screening
Ragland [34]	Single-center, single-arm, uncontrolled study	USA	Setting: Outpatient clinic Sample size (n = 50)	Screening	Positive screening n = 21 (42%) Screening scaled used: BDI-II No follow-up provided by pharmacists
Hare [35]	Multi-center, single arm, uncontrolled study	USA	Setting: community pharmacy Sample size: (n = 18)	Screening	Positive screening n = 3 (17%) Screening scale used: HANDS Referred n = 3 (17%) Followed-up by the pharmacist n = 3 (17%) Recommendations provided by the pharmacist n = 6 (33%)
Knight [36]	Multi-center, single arm, uncontrolled study	USA	Setting: Primary care clinic Sample size: (n = 45)	Screening	Positive screening: 16 out of 33 patients (48%) without a previous diagnosis of depression Screening scale used: Zung-SDS No interventions were reported following screening
Bosman [37]	RCT Followed by an economic evaluation	The Netherlands	Setting: community pharmacy Sample size: (n = 88)	Education and monitoring	Main outcomes measured: Adherence rate, patient satisfaction, cost-effectiveness, and depression symptoms using the SCL At 6 months there were no statistically significant differences between the two groups in adherence rates or symptom improvement The incremental cost-effectiveness ratio for coaching and education by pharmacists compared with usual care was €149 per 1% improvement in adherence and €2550 per point improvement in the SCL depression mean item score
Knox [38]	Single center, single arm, uncontrolled study	USA	Setting: Community pharmacy Sample size: (n = 25)	Screening	Positive screening n = 1 (4%) Screening scales used: SDS Referred n = 2
Rickles [39]	RCT	USA	Setting: Community pharmacy Sample size: (n = 60)	Education and monitoring	Main outcomes measured: Adherence rate and KAB Adherence methods used: percentage of missed doses KAB assessed using patient feedback At 3 months, higher rates of adherence in the intervention than in the control group were reported (70% vs. 51%) At six months, the intervention group had a significantly lower rate of missed doses (30% vs. 49%, * p* = 0.090) Patient feedback was significantly higher in the intervention group than in the usual care group Patients who gave more feedback to the pharmacist had significantly better antidepressant knowledge (*p* < 0.05), more positive antidepressant beliefs (*p* < 0.05), and more positive perceptions of progress (*p* < 0.001) at the end of the 3-month intervention period
Crockett [40]	RCT	Australia New south wales	Setting: Community pharmacies (32) Sample size (n = 106 patients)	Education and monitoring	Main outcomes measured: Adherence rate and KAB Scales used: K10, DAI and patient self-report Adherence: No difference between control and intervention groups (95% vs. 96%, respectively, at two months) as self-reported by the patients Well-being: K10 scores (out of a maximum of 50 points) decreased significantly from baseline to two months in both groups: by 4 points in the control group (*p* < 0.0001) and by 4.7 points in the intervention group (p ≤ 0.001) The mean DAI score increased significantly in the intervention group from 16.6 to 19.8 (*p* = 0.014) with no statistically significant change in the control group (19.6–21.1, * p* = 0.111)
Al-Saffar [41]	Randomized, two-arm single center Uncontrolled study	Kuwait	Setting: Hospital outpatient clinic Sample size: (n = 270)	Education and monitoring	Main outcome measured: Adherence rate Adherence methods used: patient self-report, clinic attendance, and tablet counting Good medication adherence at 2 and 5 months was more common in patients who were given a PIL (OR = 3.0, CI = 1.7–5.3) or a PIL plus counselling (OR = 5.5, CI = 3.2–9.6) Clinic attendance was more likely when patients had received a PIL (OR = 2.1, CI = 1.3–3.2) or a PIL plus counselling (OR = 3.2, CI = 2.1– 4.9)
Brook [42]	RCT with a 6-month follow-up	The Netherlands	Setting: Community pharmacy Samples size: (n = 147)	Education and monitoring	Main outcome measured: Adherence rate and improvement in depression symptoms Adherence methods used: pills consumed per day and adherence improvements Adherence rate improved by 17% (90% vs. 73%, CI = 5.1–28.9) No statistically significant improvement in depressive symptoms using SCL-13 scale
Rickles [43]	RCT	USA	Setting: Community pharmacies Sample size: 63 patients	Education and monitoring	Main outcomes measured: KAB, frequency of feedback to pharmacist, improvement in depressive symptoms Scales used: BDI-II,, FPFP Both groups showed significant reductions in symptoms from baseline to the end of the 3-month intervention period (*p* < 0.001). The percentages of patients experiencing at least a 50% improvement in BDI-II scores were not significantly different in the two groups Drug knowledge and belief in the medicine were rated higher by patients in the intervention group (75% vs 48%). In which IG had a significantly better overall knowledge score (*p* < 0.05) The mean of the total (FPFP) score was substantially higher in the IG than in the usual care group (23 vs 11, * p* < 0.001) at 3 months
Brook [44]	RCT with a 6-month follow-up	Netherlands	Setting: Community pharmacies Sample size: (n = 151 patients)	Education and monitoring	Main outcome measured: Improvement in depressive symptoms Scales used: SCL-90 Significant improvement in depression symptoms over 6 months in the intervention group who had three coaching contacts and watched videotape (baseline mean item score = 3.1 vs. 6-month item score 1.9) compared to the control group (baseline mean item score 2.8 vs. 6-month item score = 2.1) * p* = 0.027
Bultman [45]	Prospective cohort multi-center, uncontrolled study	USA	Setting: Community pharmacy Sample size: (n = 100)	Education and monitoring	Main outcomes measured: Adherence rate and patient satisfaction Adherence rate: 76% within 3 months. 24% improvement from baseline among patients receiving their first antidepressant Medication discontinuation rate: 24% within 3 months Treatment adherence and patient satisfaction had a positive correlation with the intervention (r = 0.49)

n, number of cases; PHQ-9, Patient Health Questionnaire-9; RCT, Randomized controlled Trial; BDI-II, Beck Depression Inventory-II; GDS, Geriatric Depression Scale; PCPs, Primary Care Providers; WHO-5, Five Well-Being Index; HCP, Health Care providers; GP, General practitioner; CES-D, Centre for Epidemiologic Studies-Depression Scale; QOL, quality of life; SF-36, 36 item Short Form Health Survey; TAU, Treatment As Usual; PHQ-2, Patient Health Questionnaire-2; HRQOL, Health-Related Quality Of Life; KAB, Knowledge Attitudes and Beliefs; NNT, Number Needed to Treat; QALYs, Quality Adjusted Life-Years; IG, Intervention Group; DIT, Distress and Impact Thermometer; HANDS, The Harvard Department of Psychiatry National Depression Screening Day Scale; SDS, Zung Self-Rating Depression Scale; SCL-13, self-reported13-item depression subscale; K10, Kessler Psychological Distress Scale; DAI, Drug Attitude Index; PIL, Patient Information Leaflet; OR, Odds Ratio; CI, Confidence Intervals; FPFP, Frequency of Patient Feedback to Pharmacist; SCL-90, The self-rating 90-items (Hopkins) Symptom Checklist

#### Pharmacist-led comprehensive depression management strategies

As summarized in Table 3, all the studies reporting on pharmacist-led comprehensive depression management strategies showed a significant impact on patient outcomes, including improvement in depression severity, reduction in antidepressant side effect occurrence, timely detection and management of potential or actual drug-related problems (DRPs), enhancement of patients’ quality of life, and promotion of adherence [46–49]. In the majority of these studies (3/4, 75%), pharmacists employed a comprehensive medication therapy management approach when providing pharmaceutical care to people with depression [28–46].

**Table 3:** Characteristics of studies evaluating pharmacist-led comprehensive depression management strategies

References	Study design	Country of the study	Setting, sample size	Elements of pharmaceutical care	Reported results/outcome measures
Polomoff [46]	RCT	USA	Setting: community pharmacy Sample size: 188 patients	MTM included: Medication review Personal medication record Medication action plan Intervention or referral, documentation, and follow-up DTP identification and resolution	Main outcomes measured: Changes in forgetness, barriers, and beliefs as predictor of DTP resolution and improvement in medications related outcomes Scales used: HSCL BMQ, MVTAS Changes in forgetness: At 15 months, there was a significant difference (*p* = 0.030) such that the average score in the EWS + MTM arm was 0.26 units lower than the average of the other 2 groups (social services and EWS) Trajectory for barriers to medication: At 15 months, there was no significant difference between the EWS + MTM arm vs. other groups (*p* = 0.338) BMQ necessity and concern: No discernable pattern was evident at 15 months (necessity, * p* = 0.533; concern, * p* = 0.873) The average number of DTPs per patient in the EWS MTM arm was 6.57 and 84% of DTPs were resolved
Gomes [47]	Pre-post interventional study	USA	Setting: Hospital Sample size: (n = 20)	MTM (Dader method) that included: Health assessment Medication history Recognize and address any PRM raised by patients	Main outcomes measured: Improvement in depressive symptoms and quality of life Scales used: PHQ-9, SF-36 There was a statistically significant decrease in depression symptoms post intervention (*p* = 0.0001) There was a statistically significant improvement in all the QOL domains post intervention (*p* < 0.05)
Marques et al. [48]	RCT Conventional treatment (CG) vs. Interventional (IG)	Brazil	Setting: Outpatient clinic and patient homes Sample size: 68 patients	MTM (Dáder Method) that included: Identify DRPs and DNOs Perform individualized care plan Assess the results of the intervention	Main outcome measured: Improvement in symptoms severity and patient satisfaction with the follow-up Scale used: BDI The median reduction in depressive symptoms as per the BDI scores was 2.5 points in CG and 3.5 points in the IG Cases of severe depression were reduced by 80% in the IG compared to 60% in the CG. Cases of moderate depression were reduced by 53.4% in the IG vs. 7.7% in the CG A total of 57 DNOs were found in 88% of the patients, and the pharmacist's help resolved 64.9% of them Patient satisfaction: The outcomes shown satisfaction of the IG and CG The majority of patients (95.8%) indicated that they would continue seeing the pharmacist or receiving additional visits from the pharmacist
Canales [49]	Two phases Control (usual care) and Experimental (intensive PC) groups	USA	Setting: psychiatric hospital Sample size: 93 patients	Intensive PC included: Baseline assessments and weekly reviews Medication histories and daily review of drug administration records Pharmacotherapy recommendations ADRs monitoring Weekly medication education classes Counseling patients before discharge	Main outcomes measured: Adherence rate, clinical improvement, and adverse events Scales used: BPRS, CGI, HAM-D, AIMS, Barnes, and Simpson-Angus Significant improvement in adherence rate (up to 27%) Significant improvement in BPRS and CGI scores at discharge in the experimental group than in the control group (*p* < 0.001) Significant improvement in depression symptoms in the experimental vs. the control group, as per the HAM-D scores (65% vs 9%, respectively; * p* = 0.003) Adverse events: Positive mean percent changes in the AIMS, Barnes, and Simpson-Angus scores in the experimental group (*p* < 0.024, * p* < 0.042, * p* < 0.002, respectively)

RCT, Randomized controlled Trial; MTM, Medication Therapy Management; DTP, Drug Therapy Problems; HSCL, Hopkins Symptom Checklist; MVTAS, Modified Version of the Therapeutic Alliance Scale; BMQ, Beliefs about Medicine Questionnaire; EWS, Eat Walk Sleep; PRM, Potential Problems Related to Medicines; PHQ-9, Patient Health Questionnaire; SF-36, 36 item Short Form Health Survey; QOL, quality of life; IG, Intervention group; CG, Control Group; DNOs, Drug-related Negative Clinical Outcomes; DRP, Drug Related Problems; BDI-II, Beck Depression Inventory-II; PC, Pharmaceutical care; ADRS, Adverse Drug Reactions; BRPS, The 18-item Brief Psychiatric Rating Scale; CGI, Clinical Global Impressions scale; HAM-D, 1 Hamilton Psychiatric Rating scores for mood disorder; AIMS, The Abnormal Involuntary Movement scale

#### Pharmacists’ collaborative care practices in depression management

As summarized in Table 4, the majority of the studies reporting on pharmacists’ collaborative care practices in depression management, such as personalized care planning or SDM, were randomized clinical trials (RCTs) comparing the intervention (collaborative care) versus usual care. The majority of the studies in this category (10/12, 83%) reported that participants in the intervention group had positive depression treatment outcomes as a result of the intervention [50–60]. Compared to usual care, participants in the intervention group were more likely to have a significant decrease in symptoms or in depression severity [50–52, 54, 55, 60], improved antidepressant adherence rates [53, 55, 57–59] and a higher satisfaction with the treatment plan or their quality of life [53, 55, 58].

**Table 4:** Characteristics of the studies evaluating pharmacists’ collaborative care practices in depression management

References	Study design	Country of the study	Setting, sample size	Description of collaborative care provided	Reported results/outcome measures
Kanwal [50]	RCT IG vs. UCG	USA	Setting: Clinics Sample size: (n = 263)	Stepped-care model was followed by pharmacists when providing services to the IG but not to the UCG. The model consisted on: DCM monitoring Pharmacotherapy recommendations (based on site specific guidelines) by clinical pharmacist after review of depression treatment history Combination pharmacotherapy and specialty mental health counseling Referral to specialty mental health services Collaborative care team members: Depression care manager, pharmacist, and psychiatrist	Main outcome measured: Improvement in depression symptoms, patient satisfaction, and adherence rate Scales used: SCL-20, SF-12 There was a significant improvement in depression severity (*p* = 0.002), remission (*p* = 0.004) at 12 months in the IG vs. the UCG The patient satisfaction with care, antidepressant adherence rate and the SF-12 (physical component) scores were not statistically different in the IG vs. the UCG (*p* = 0.87, * p* = 0.12, * p* = 0.96, respectively)
Greene [51]	Retrospective review of patients’ electronic health records before (UCG) and after (IG) implementation of a depression protocol	USA	Setting: Clinic Sample size: (n = 49)	The IG consisted of patients diagnosed with depression who received care by MDT before and after implementation of a collaborative care protocol. If patients were prescribed an antidepressant, the pharmacist would provide: Initial education on the pharmacological management Conduct follow up assessments, including medication adherence, assessment of side effects, assessing self-management objectives (improved coping), and relative efficacy (from the patient's perspective), and re-evaluation of suicide risk Dosage modification in consultation with other members of the MDT The UCG consisted of patients diagnosed with depression prior to implementing the protocol Collaborative care team members: Behavioral health consultant, pharmacist, primary care physician, consulting psychiatrist and psychiatric nurse practitioner	Main outcome measures: Improvement in depression symptoms (depression severity, remission rate) Scales used: PHQ-9 The mean reduction in PHQ-9 score was 7.06 points greater IG than in the UCG (*p* = 0.001, 95% CI, 4.3–9.8) Patients in the IG (94%) were more likely than those in the UCG (50%) to experience a drop in PHQ-9 of 3 or more (*p* = 0.001) More patients in the IG (62.8%) than in the UCG (14.3%) had a response to treatment, as indicated by a 50% or more reduction in the PHQ-9 score (*p* = 0.004) None of the patients in the UCG experienced remission, as opposed to 11 in the IG (31.5%) (*p* = 0.021) After a 2-week telephone follow-up, 57% of the UCG patient’s vs 80% in the IG self-reported improvement, respectively (*p* = 0.152)
Binakaj 2016 [52]	RCT IG vs. UCG	Bosnia and Herzegovina	Setting: Hospital Sample size: (n = 48)	Psychiatrist in collaboration with pharmacists monitored monthly patients in the IG for a period of six months, as part of a supportive pharmaceutical care program provided by the pharmacist, which consisted on: Patient education on antidepressants Medication monitoring including side effects and interactions with other drugs Implementation of evidence-based treatments for depression Patients in the UCG only made standard appointments with a psychiatrist Collaborative care team members: Pharmacist, psychiatrist, medical doctor	Scales used: Unspecified questionnaire with QoL and depression symptoms-related questions Responses to the questionnaire were indicative that patients in the IG had a better response to treatment than patients in the UCG: eg, 29% more patients in the IG reported to “rarely experiencing depressive symptoms” over the 6-month study period
Aljumah [53]	RCT IG vs. UCG	KSA	Setting: Hospital Sample size: (n = 139	The UCG received usual pharmacy services and the IG received usual pharmacy services plus pharmacist involvement after referral from the psychiatry department following a protocol that included a SDM approach Pharmacists in the IG applied SDM competency framework, with the aim of increasing patients' participation in SDM by evaluating their attitudes and knowledge about antidepressants Collaborative care team members: Pharmacist and MDT from the psychiatry department	Main outcomes measured: Adherence rate, improvement in depression symptoms, patients’ beliefs, QoL, and patient satisfaction Scales used: MMAS, TSQM, BMQ, MADRS, EQ-5D, and OPTION scale A statistically significant improvement in medication adherence (as per MMAS score) was achieved in the IG compared to patients in the UCG after 6 months (*p* < 0.0001) A statistically significant patient satisfaction (as per the TSQM score) was observed after 6 months in the IG compared to UCG (*p* < 0.0001) A positive change in the patients’ beliefs about medicines was achieved in the IG after 6 months (as per the BMQ scores) No significant improvement in the severity of depressive symptoms was observed in the IG at the end of 6 months (*p* = 0.897) No results were reported in regards to SDM (as per the OPTION scale) No significant improvement in HRQoL (as per the EQ-5D score) (*p* = 0.722)
Pyne [54]	RCT IG vs. UCG	USA	Setting: HIV clinics at psychiatry hospital Sample size: (n = 360)	The collaborative team employed a stepped-care strategy that included SDM. Along with DCM monitoring, the 5-step strategy included: Watchful waiting Depression care team treatment recommendations (counselling or pharmacotherapy, depending on participant preference) Pharmacotherapy recommendations following clinical pharmacist examination of depression history Combination pharmacotherapy and specialty mental health services Collaborative care team members: A registered nurse was the depression care manager, a pharmacist, and a psychiatrist	Main outcomes measured: Improvement in depression severity and number of depression free days, adherence rate, QoL, and patient satisfaction Scales used: SCL-20, PHQ-9, SF-12 Compared to the UCG, patients in the IG were more likely to report treatment response (33.3% vs. 17.5%; odds ratio, 2.50; 95% CI, 1.37–4.56) and remission (22.0% vs. 11.9%; 2.25; 1.11–4.54) at 6 months, but not at 12 months Participants in the IG reported more days without depression over the course of a year compared to UCG (β = 19.3; 95% CI, 10.9–27.6; * p* = 0.001) No significant difference in the adherence rate between IG (76.3%) and UCG (85.0%) at 6 months (*p* = 0.27) There was no outcomes reported for patient satisfaction
Fortney [55]	RCT IG vs. UCG	USA	Setting: Community based outpatient clinics Sample size: (n = 395)	Both IG and UCG sites received provider education (via interactive video and website) and patient education (via mail and website) The IG underwent depression treatment using a 3 stepped-care model for up to 12 months that included: Watchful waiting Depression care team treatment recommendations The pharmacist obtained a medication history and recommended pharmacotherapy to PCPs in an electronic progress note The pharmacist offered non-scripted medication management over the phone to patients who were struggling with non-adherence or who were experiencing severe side effects For non-respondent patients tele-psychiatry consultation before making additional treatment recommendations to the patient's PCP Collaborative care team members: Tele-psychiatrist, off-site depression nurse case manager, off-site clinical pharmacist, and PCP	Main outcomes measured: Antidepressant prescribing, medication adherence, treatment response and remission, and patient satisfaction Scales used: PHQ-9, DUSOCS, SCL-20, SF12V, Mini International Neuro-psychiatric Interview No significant difference in the likelihood of having an active prescription between the groups at twelve months (OR = 1.3, * p* = 0.40) Patients in IG (76.4%) had significantly greater medication adherence than those in UCG (66.2%) at 12 months (OR = 2.7, * p* < 0.01) There was no significant difference in response between IG and UCG (53% vs 51%) at 12 month (*p* = 0.18), but significant remission rate between IG and UCG at 12 month (24% vs 12.7%, respectively, * p* = 0.02) Patients in IG (70.9%) were significantly more satisfied with the treatment at 12 months compared to patients in the UCG (61.4%), * p* = 0.03)
Capoccia [56]	RCT IG (enhanced care) vs. UCG	USA	Setting: Primary care clinic Sample size: (n = 74)	IG received enhanced care by a clinical pharmacist or pharmacy resident, who delivered multifaceted interventions in collaboration with PCPs and a psychiatrist. The enhanced care consisted on: Initial support and patient education were provided At each contact, depressive symptoms and medication-related concerns were addressed Ongoing monitoring, medication dosage adjustments and management of adverse effects as needed Access to patient assistance programs was facilitated Patients in the UCG were encouraged to use available resources (PCPs, pharmacists, nurses, and mental health providers), as needed Collaborative care team members: family physicians and psychiatrist, residents in family medicine and psychiatry, behavioral scientist, mental health intern, social worker, physician assistants, nurses, clinical pharmacist, pharmacy resident	Main outcomes measured: Adherence rate, patient satisfaction, and clinical improvement in depression symptoms Scales used: SCL-20, SCID, SF-12 and DSM-IV There was no difference between IG and UCG in adherence rate at the 12-month follow-up (59% vs 57%, respectively, * p* = 0.91) No significant difference in satisfaction with depression care between IG and UCG at the 12-month follow-up (80% vs. 77%, respectively, * p* = 0.19) The number of patients with a 50% or more decrease in SCL-20 score during the study period did not differ between groups (*p* = 0.39)
Adler [57]	RCT IG vs. UCG	USA	Setting: Primary care clinic Sample size: (n = 533	The IG patients received consultation in person and over the phone by a clinical pharmacist, who assisted them as well as the patients’ PCP with medication selection, dosage, and schedule in accordance with depression guidelines. Patients in the IG received personalized care services provided by clinical pharmacist, which consisted on: Complete medical and medications history assessment Assessment and identification of DRPs Evaluation on safety and efficacy of medications Patient education Facilitate communication between patient and the other PCP Patients in the UCG received standard PCP care Collaborative care team members: PCPs (internists, family practitioners, and nurse practitioners), and clinical pharmacist	Main outcomes measured: Adherence rate and improvement in depression severity at 6 months Scales used: mBDI The adherence rate improved by 11% at 6 months in IG compared to UCG respectively (57.5 vs 46.2, * p* < 0.025) Antidepressant use was higher in the IG at 6 months compared to UCG respectively (32.3% vs. 10.9%, * p* < 0.001) changes in the mBDI scale were not statistically significant between the IG and UCG at 6 months respectively (17.7 vs 19.4, * p* = 0.16)
Finley [58]	RCT IG vs. UCG	USA	Setting: Medical Centre Sample size: (n = 120)	The IG was referred to a clinical pharmacist by internal medicine physician or psychiatrist. The UCG received usual pharmacy services For the IG, the clinical pharmacist was responsible for: -Drug therapy management and treatment follow-up through phone calls at weeks 1, 2, 4, 10, and 16 Drug monitoring Patient education Prescribing and medication changes under protocol Collaborative care team members: Clinical pharmacists, internal medicine physician, psychiatrist	Main outcome measures: Adherence rate, clinic visits, drug cost, clinical and functional outcomes, patient satisfaction Scales used: BIDS, WSDS, MPR The adherence rate increased by 19% (67% in IG vs 48% in UCG) * p* = 0.038 Clinic visits decreased by 15% in IG vs 2% in UCG (*p* = 0.14) Patients in the IG were more satisfied with treatment compared to the usual care group (*p* < 0.05) Drug cost increased by 42% in the IG No significant difference between the two groups in improvement of depressive symptoms Patients in the IG were more likely to change antidepressants during the study period 19% in the IG vs. 4% in the usual care group, * p* = 0.016
Finley [59]	Non-RCT Cohort study IG vs. CG	USA	Setting: Primary care clinic Sample size: (n = 220)	Patients in the IG diagnosed with depression, and started on an antidepressant, were referred by PCPs to the practice model HMO. Clinical pharmacy specialists then coordinated the patients' follow-up for six months via a mix of scheduled office visits and phone calls, in close collaboration with psychiatric liaisons The CG was identified retrospectively through a comprehensive review of automated pharmacy refill records for patients starting on antidepressants during the same nine-month time frame, but who were not referred to the pharmacist Collaborative care team members: Physicians, nurse practitioners, pharmacist, clinical pharmacist specialist	Main outcomes measures: Adherence rate, clinic visits and patient satisfaction Scales used: CGI and MPR At the end of the study, the adherence rate improved by 15% in the IG compared to the UCG (81% vs 66%, * p* = 0.0005) Overall, medication adherence was higher in the IG compared to the UCG (76% vs. 53%, * p* = 0.008) Clinic visits to PCPs declined for patients in the IG compared to the UCG (39% vs. 12%, * p* = 0.029) indicative of less resource utilization Medication switch rates were higher in the IG vs. the UCG (24% vs 5%, * p* = 0.0001) Six-month MPRs were significantly greater for the IG vs. the UCG (0.811 vs 0.659, * p* < 0.005) Patients in the IG were more satisfied (*p* < 0.05) with the treatment at the HMO
Finley [60]	RCT IG vs. UCG	USA	Setting: Outpatient clinic Sample size: (n = 114)	Patients in the IG diagnosed with depression, and started on an antidepressant, were referred by PCPs to a clinic guided by a clinical pharmacist, who had prescribing authority for dose titrations or addition of ancillary medications. The clinical pharmacists: Educated patients about depression, pharmacotherapy, importance of medication adherence, and management of side effects Notified the PCP about patient progress and contacted psychiatry specialist as needed The UCG received their usual care by providers Collaborative care team members: clinical pharmacist, clinicians from psychiatry and internal medicine department, social worker, nurse specialists and psychologist	Main outcomes measured: Improvement in depression symptoms, patient satisfaction Scales used: IDS, CGI, WSDS Patients in the IG who received a 4-week medication trial, 76% were considered a therapeutic responder and 86% achieved remission. The percent responders among the UCG was not reported Average daily doses of antidepressants were lower in the IG, suggesting that patients receiving intensive case management may respond to lower doses of antidepressants
Schmidt [61]	RCT Intervention vs. control nursing homes	Sweden	Setting: Nursing homes Sample size: (n = 1854)	In the intervention nursing homes, prescription lists for each resident were assessed one month before and one month after the 12-month MDT intervention, in which psychotropic medication were assessed in terms of quantity and quality to reduce the use of medications not recommended to be used in this population, as defined by the SMPA guidelines Collaborative care team members: physicians, pharmacists, staff nurses, and nursing assistants	Main outcomes measured: Quantity and quality of psychotropic medications Significant higher average number of medications prescribed (*p* = 0.02) and percentage of residents who had therapeutic duplication (*p* = 0.04) in the control nursing homes TCA use decreased by 59% (*p* = 0.001) in the experimental nursing homes compared to 34% (*p* = 0.002) in the control group SSRI usage increased by 315% (*p* = 0.001) in the control nursing homes and by 584% (*p* = 0.001) in the intervention nursing homes In the intervention nursing homes, the percentage of residents taking any antidepressant rose from 19 to 25% (*p* = 0.007), while in the control nursing homes, it rose from 16 to 22% (*p* = 0.001)

vs., versus, IG Intervention Group; UCG, Usual Care Group; DCM, Depression Care Management; SCL-20, Hopkins Symptom Checklist-20; SF-12, Short Form 12 item Health Survey; MDT, Multidisciplinary team; PHQ-9, Patient Health Questionnaire-9; CI, Confidence Interval; QoL, Quality of life; SDM, Shared decision making; MMAS, Morisky Medication Adherence Scale; TSQM, Treatment Satisfaction Questionnaire for Medication; BMQ, Beliefs About Medicines Questionnaire; MADRS, Montgomery–Åsberg Depression Rating Scale; EQ-5D, European Quality of Life 5 Dimensions 5 Level Version; OPTIONS, Observing Patient Involvement in Decision-Making Scale; HRQOL, Health-Related Quality Of Life; PCPs, Primary care providers; DUSOCS, Duke Social Support and Stress Scale; DSM-IV, Diagnostic and Statistical Manual of Mental Disorders, Fifth Edition; mBDI, Modified Beck Depression Inventory; DRP, Drug-related problem; BIDS, Brief Inventory for Depressive Symptoms; WSDS, Work and Social Disability Scale; MPR Medication possession ratio; HMO, Health maintenance organization; CGI, Clinical Global Impression; IDS, Inventory for Depressive Symptomology; SMPA, Swedish Medical Product Agency

### Barriers to effective management of depression

The most common barrier to effective depression management reported in the studies was time constraints [32, 41, 46]. The lack of privacy and confidentiality associated with the conventional layout of community pharmacies was another reported reason hindering the pharmacists’ ability to provide depression care [32, 41, 56]. Mental health stigma and low patient awareness of the pharmacist’s role was reported in some studies as a barrier for patients to seek care from community pharmacists [20, 32, 56]. Some studies reported difficulties in patient follow-ups [20, 31, 46] and in collaborating with other healthcare providers [31, 66]. When studies with limited impact were examined, other barriers were identified such as operational constraints [37], insufficient duration of intervention implementation [40], patients’ non-adherence to the intervention [42], and lack of intervention documentation [56].

### Pharmacist-led management of depression in the Arab region

Only 3 studies were found reporting on pharmacists’ interventions in the management of depression in Arab countries [23, 41, 53]. Two of these studies reported on “pharmacist-led specific/single depression interventions/management strategies” (category 1) [23, 41] and one on “collaborative care practices in depression management” [53]. These studies reported positive outcomes as a result of the pharmacist intervention, such as improvement in depression symptoms [23], improvement in adherence rates [41]. In the study conducted in Saudi Arabia [53] collaborative depression care provided by pharmacists using a SDM model of practice resulted in significant improvement in the treatment adherence rate (*p* < 0.0001), treatment satisfaction (*p* < 0.0001), and KAB about depression and its treatment (*p* = 0.021) compared to patients receiving standard care [53].

## Discussion

### Statement of key findings

The present scoping review yielded a total of 40 studies discussing pharmacist role in depression care and management. More than half of the studies focused on pharmacist-led specific/single interventions. Pharmacist interventions included screening, improvement of adherence rate, and education/monitoring. The studies identified in this review showcased a positive impact on patient outcomes like adherence and satisfaction. Nonetheless, reported barriers to effective management reported included challenges with time constraints, privacy, mental health stigma, follow up, and collaboration with other professionals. Interestingly, only three studies within this review focused on pharmacist-led interventions in the Arab region. This comprehensive overview underscores the diversity, depth, and potential of pharmacist strategies in depression care, emphasizing the imperative need to address barriers to maximize positive outcomes. It also sheds light on the scarcity of such evidence in the Arab region, warranting further research in this area.

### Interpretation

The studies included in this scoping review provide evidence of the diversity and depth of pharmacists’ strategies in depression care. The review also provides information on the impact of these strategies, either pharmacist-led or in collaboration with other members of the healthcare team, on depression treatment outcomes. The majority of the studies included reported on pharmacist-led depression screening and patient education/monitoring strategies, demonstrated a positive effect on the overall management of depression, including referral of patients for more thorough assessments or treatment initiation, an overall improvement in adherence to antidepressants and in patients’ KAB about depression and its treatment. These findings are important for supporting a continued expansion of the pharmacist’s role in depression care. Despite depression posing a significant burden on society, many people do not seek help for their symptoms and treatment is often not adequate [6]. Depression screening by pharmacists is a strategy that has shown positive results and should be a commendable role for pharmacists in depression care. The results of a systematic review on pharmacist-led depression screening services also showed the intervention resulted in positive outcomes, such as early identification and referrals of people at risk of depression to appropriate health services [62].

Another important outcome of pharmacists’ depression care services reported in the studies included in this scoping review is in relation to improvement in the treatment adherence rates. These results are in line with those from systematic reviews which showed that patient counseling and treatment monitoring conducted by pharmacists can improve adherence to antidepressant medications [63–65]. Although a previous systematic review reported inconclusive findings in regard to the efficacy of pharmacist-based depression management on improving depression symptoms when compared to usual care [66], considering that non-adherence to antidepressants is high [67, 68], by improving treatment adherence through pharmacist’s interventions, patients are more likely to experience less symptoms of depression. Adherence to antidepressant treatment is not only essential in achieving remission and restoring the patient’s previous levels of functioning, but also in preventing re-occurrence. When dispensing medications, pharmacists are in a suitable position to educate and monitor patients on their medications, but also to collaborate with other healthcare providers to support patients during their treatment, monitor progress and promote the importance of medication adherence.

For the most part, studies classified under category 2 and 3 reported more clinical outcomes such as depression symptom improvement or a decrease in the depression severity using validated psychometric tools, and were evaluated against the outcomes in a comparator group receiving usual care. The majority of these strategies demonstrated a significant difference in decreasing depression severity, reducing troublesome antidepressant side effects, timely detection and management of potential or actual DRPs, and enhancement of patients’ quality of life. Despite these positive results, it is important to bear in mind that quality assessment of the included studies was not done, and thus, caution in the interpretation of these results is recommended. Future studies should use more robust study designs, more specific and sensitive evaluation measures and involve longer follow up periods [9, 66].

The notable difference in the number of studies undertaken in North America compared to those in other parts of the world, and particularly in the Arab region, is possibly related to the vast differences in the scope of pharmacy practice in these two regions. It is well known that pharmacists in many states in the USA and provinces in Canada are allowed to refill, modify, and even prescribe medications under a protocol or collaborative agreements, which may facilitate a more direct patient care role for pharmacists when monitoring patients on depression treatment [69]. Limited public awareness about the community pharmacists’ role beyond those related to dispensing medications may also contribute to this paucity in the provision of more advanced or clinically focused community-based services observed in most Middle Eastern countries [70, 71]. There is also a wide gap in pharmacy practice across the different healthcare settings. Hospital pharmacists in the Middle East often possess an advanced degree and tend to have a higher level of practice compared to that of community pharmacists [72]. However, even in the hospital setting, pharmacists are challenged by other mental healthcare providers. In a recent survey to physicians and nurses working in a psychiatric hospital in Qatar, mostly positive perceptions and expectations from clinical pharmacists were reported, although traditional clinical pharmacy services were more favorably viewed than those associated with advanced clinical roles such as prescribing and pharmacist-led medication management clinics [73]. As such, it is important that countries within the Arab region set strategies in support of an expanded pharmacist scope of practice, not only to improve their professional image, but also to improve early and sustainable access to depression care.

Barriers for the provision of pharmacist-facilitated depression care reported in the studies included in this scoping review are similar to those in relation to implementation of any new pharmacy service, such as time constraints and training needs [74, 75]. In addition to that, barriers specifically related to mental health, such as pharmacists’ attitudes, stigma and communication skills have been highlighted in these studies. Mental health stigma makes patients and pharmacists afraid to discuss mental health disorders openly [30, 46, 57, 74, 76]. Some countries are exploring strategies to improve mental health literacy among pharmacists to support them in the provision of mental health services. For instance, in recent studies in Australia where community pharmacists were interviewed and surveyed, participants have emphasized on the significance of mental health training including continuous professional education and the Mental Health First Aid (MHFA) for high-quality late-life and perinatal depression screening [74, 75]. The MHFA course is an internationally recognized training program that helps front-line healthcare professionals to address how to identify, understand and respond to signs of mental illnesses and substance use disorders [77]. This type of training is also becoming increasingly important in pharmacy education [78]. Strategies like this can not only decrease mental health stigma, but also help pharmacists to improve their communication skills and gain confidence for engaging in mental health service provision. Furthermore, pharmacists’ involvement in mental health awareness campaigns in collaboration with other members of the mental health team, such as the World Mental Health Day organized worldwide by the WHO [79], can increase public awareness not only about mental health overall, but also on the role of the pharmacist. These reported barriers, whether in studies with positive or limited intervention impact, offer insight into the potential challenges and strategies to overcoming them when implementing pharmacist-led interventions as part of depression care. Importantly, lessons learned from identified studies include the importance of intervention integration within operations so as not to disrupt usual practice, implementation of the interventions for a sufficient time before evaluation, and adequate patient education on the intervention to ensure adherence.

### Strengths and weaknesses

This scoping review offers synthesized evidence from the literature related to pharmacist-led or and collaborative strategies in depression management, classified by the breadth of the pharmacist intervention. We believe this classification facilitates a more practical analysis of the wide scope of pharmacy practice in depression care across the globe and their associated outcomes. An additional study limitation is that the manual search yielded a greater number of studies compared to the screening search. Nonetheless, the measures to overcome this limitation in fact present a particular strength of this scoping review, as a a thorough review of the references included in previous narrative and systematic reviews on the research topic was conducted as a supplemental manual search. Conference abstracts, protocols, book reviews, opinion pieces, and editorial reviews were not included in this study. As such, it is possible that some studies published in non-peer-reviewed journals or in conference proceedings were not captured.

### Further research

Future studies are needed to investigate pharmacists’ roles in depression and mental health, especially in the Arab region. Studies can utilize experimental methods to evaluate the efficacy and long-term impact of pharmacist-led interventions on patient quality of life and clinical outcomes. The barriers identified and strategies to overcome them should also be explored to ensure maximum effectiveness of said interventions. Furthermore, it is crucial to study policies and training initiatives employed or needed to support an expanded scope of practice for pharmacists in depression care.

## Conclusion

This collection of evidence confirms that pharmacists can play an important role in supporting people experiencing depression and in improving depression treatment outcomes. Pharmacist-led focused interventions such as depression screening, education and treatment monitoring have resulted in early identification, referrals and improved treatment adherence. More comprehensive pharmaceutical care and collaborative depression management interventions have also shown similar positive patient outcomes; however, more robust studies are needed with longer follow-up periods that evaluate their long-term sustainability. Nevertheless, this scoping review can be used as preliminary evidence with stakeholders advocating for an expanded scope of practice for pharmacists in mental health, particularly in countries within the Arab region.

## Supplementary Information

Below is the link to the electronic supplementary material.

Supplementary file1 (DOCX 17 KB)

## References

[1] James SL, Abate D, Abate KH, et al. Global, regional, and national incidence, prevalence and years lived with disability for 354 diseases and injuries for the burden of disease study 2017. Lancet. 2018;392(10159):1789–858. 10.1016/S0140-6736(19)30427-1. 30496104 10.1016/S0140-6736(19)30427-1 PMC6227754

[2] World Health Organization. Depression [internet]. 2021. Available from: https://www.who.int/news-room/fact-sheets/detail/depression#. Accessed 07 Feb 2023.

[3] Institute for Health Metrics and Evaluation. New Global Burden of Disease analyses show depression and anxiety among the top causes of health loss worldwide, and a significant increase due to the COVID-19 pandemic [internet]. 2021. Available from: https://healthdata.org. Accessed 07 Feb 2023.

[4] Lépine JP, Briley M. The increasing burden of depression. Neuropsychiatr Dis Treat. 2011;7(Suppl 1):3–7. 10.2147/NDT.S19617. 21750622 10.2147/NDT.S19617 PMC3131101

[5] Greenberg PE, Fournier AA, Sisitsky T, et al. The economic burden of adults with major depressive disorder in the United States (2010 and 2018). Pharmacoeconomics. 2021;39(6):653–65. 10.1007/s40273-021-01019-4. 33950419 10.1007/s40273-021-01019-4 PMC8097130

[6] Thornicroft G, Chatterji S, Evans-Lacko S, et al. Under-treatment of people with major depressive disorder in 21 countries. Br J Psychiatry. 2017;210(2):119–24. 10.1192/bjp.bp.116.188078. 27908899 10.1192/bjp.bp.116.188078 PMC5288082

[7] El-Den S, Collins JC, Chen TF, et al. Pharmacists’ roles in mental healthcare: past, present, and future. Pharm Pract (Granada). 2021;19(3):2545. 10.18549/PharmPract.2021.3.2545. 34621456 10.18549/PharmPract.2021.3.2545 PMC8456342

[8] McMillan SS, Kelly F, Hattingh HL, et al. The impact of a person-centred community pharmacy mental health medication support service on consumer outcomes. J Ment Health. 2018;27(2):164–73. 10.1080/09638237.2017.1340618. 28675321 10.1080/09638237.2017.1340618

[9] Rubio-Valera M, Chen TF, O’Reilly CL. New roles for pharmacists in community mental health care: a narrative review. Int J Environ Res Public Health. 2014;11(10):10967–90. 10.3390/ijerph111010967. 25337943 10.3390/ijerph111010967 PMC4211017

[10] Millonig MK. White paper on expanding the role of the community pharmacist in managing depression [Internet]. 2008. Available from: https://www.aphafoundation.org/sites/default/files/ckeditor/files/WhitePaper-PharmacistRoleManagingDepression-APhAFoundation-2009(1).pdf. Accessed 01 Dec 2023.

[11] Stuhec M, Hahn M, Taskova I, et al. Clinical pharmacy services in mental health in Europe: a commentary paper of the European Society of Clinical Pharmacy Special Interest Group on Mental Health. Int J Clin Pharm. 2023;45(5):1286–92. 10.1007/s11096-023-01643-4. 37755642 10.1007/s11096-023-01643-4 PMC10600282

[12] Melton BL, Lai Z. Review of community pharmacy services: what is being performed, and where are the opportunities for improvement? Integr Pharm Res Pract. 2017;6:79–89. 10.2147/IPRP.S107612. 29354554 10.2147/IPRP.S107612 PMC5774328

[13] Giannetti V, Caley CF, Kamal KM, et al. Community pharmacists and mental illness: a survey of service provision, stigma, attitudes and beliefs. Int J Clin Pharm. 2018;40(5):1096–105. 10.1007/s11096-018-0619-7. 29862460 10.1007/s11096-018-0619-7

[14] Sadek MM, Elnour AA, Al Kalbani NM, et al. Community pharmacy and the extended community pharmacist practice roles: the UAE experiences. Saudi Pharm J. 2016;24(5):563–70. 10.1016/j.jsps.2015.03.023. 27752229 10.1016/j.jsps.2015.03.023 PMC5059830

[15] El Hajj MS, Mekkawi R, Elkaffash R, et al. Public attitudes towards community pharmacy in Arabic speaking Middle Eastern countries: a systematic review. Res Social Adm Pharm. 2021;17(8):1373–95. 10.1016/j.sapharm.2020.11.013. 33257161 10.1016/j.sapharm.2020.11.013

[16] Ibrahim NK. Epidemiology of mental health problems in the middle east. Handbook of healthcare in the Arab World. Cham: Springer; 2021. p. 133–49. 10.1007/978-3-030-36811-1_12.

[17] Munn Z, Peters MDJ, Stern C, et al. Systematic review or scoping review? Guidance for authors when choosing between a systematic or scoping review approach. BMC Med Res Methodol. 2018;18:1–7. 10.1186/s12874-018-0611-x. 30453902 10.1186/s12874-018-0611-x PMC6245623

[18] Why a scoping review? JBI manual for evidence synthesis—JBI global Wiki. Accessed 01 December 2023. Available from: https://jbi-global-wiki.refined.site/space/MANUAL/355862553/10.1.1+Why+a+scoping+review%3F.

[19] Peters MD, Godfrey CM, Khalil H, et al. Guidance for conducting systematic scoping reviews. Int J Evid Based Healthc. 2015;13(3):141–6. 10.1097/XEB.0000000000000050. 26134548 10.1097/XEB.0000000000000050

[20] Tricco AC, Lillie E, Zarin W, et al. PRISMA extension for scoping reviews (PRISMA-ScR): checklist and explanation. Ann Intern Med. 2018;169(7):467–73. 10.7326/M18-0850. 30178033 10.7326/M18-0850

[21] Peters MDJ, Marnie C, Tricco AC, et al. Updated methodological guidance for the conduct of scoping reviews. JBI Evid Synth. 2020;18(10):2119–26. 10.11124/JBIES-20-00167. 33038124 10.11124/JBIES-20-00167

[22] Ballou JM, Chapman AR, Roark AM, et al. Conducting depression screenings in a community pharmacy: a pilot comparison of methods. J Am Coll Clin Pharm. 2019;2(4):366–72. 10.1002/jac5.1156. 10.1002/jac5.1156

[23] Alkoudsi KT, Basheti IA. Prevalence of anxiety and depression among women with polycystic ovary syndrome living in war versus non-war zone countries: a randomized controlled trial assessing a pharmacist intervention. Res Social Adm Pharm. 2020;16(5):689–98. 10.1016/j.sapharm.2019.08.027. 31420190 10.1016/j.sapharm.2019.08.027

[24] Wilson C, Twigg G. Pharmacist-led depression screening and intervention in an underserved, rural, and multi-ethnic diabetic population. J Am Pharm Assoc. 2018;58(2):205–9. 10.1016/j.japh.2017.11.001. 10.1016/j.japh.2017.11.001 29217142

[25] Kondova A, Todorova A, Tsvetkova A, et al. Screening and risk assessment for depression in community pharmacy—pilot study. J of IMAB. 2018;24(1):1928–31. 10.5272/jimab.2018241.1928. 10.5272/jimab.2018241.1928

[26] O’Reilly CL, Wong E, Chen TF. A feasibility study of community pharmacists performing depression screening services. Res Social Adm Pharm. 2015;11(3):364–81. 10.1016/j.sapharm.2014.08.013. 25438728 10.1016/j.sapharm.2014.08.013

[27] Phimarn W, Kaewphila P, Suttajit S, et al. Depression screening and advisory service provided by community pharmacist for depressive students in university. Springerplus. 2015;4:470. 10.1186/s40064-015-1259-1. 26357601 10.1186/s40064-015-1259-1 PMC4556723

[28] Klang SH, Ben-Amnon Y, Cohen Y, et al. Community pharmacists’ support improves antidepressant adherence in the community. Int Clin Psychopharmacol. 2015;30(6):316–9. 10.1097/YIC.0000000000000090. 26163876 10.1097/YIC.0000000000000090

[29] Funk KA, Hudson S, Tingen J. Use of clinical pharmacists to perform depression screening. Qual Prim Care. 2014;22(5):249–50. 25897546

[30] Rosser S, Frede S, Conrad WF, et al. Development, implementation, and evaluation of a pharmacist-conducted screening program for depression. J Am Pharm Assoc. 2013;53(1):22–9. 10.1331/JAPhA.2013.11176. 10.1331/JAPhA.2013.11176 23636152

[31] Rubio-Valera M, March Pujol M, Fernández A, et al. Evaluation of a pharmacist intervention on patients initiating pharmacological treatment for depression: a randomized controlled superiority trial. Eur Neuropsychopharmacol. 2013;23(9):1057–66. 10.1016/j.euroneuro.2012.11.006. 23219937 10.1016/j.euroneuro.2012.11.006

[32] Rubio-Valera M, Bosmans J, Fernández A, et al. Cost-effectiveness of a community pharmacist intervention in patients with depression: a randomized controlled trial (PRODEFAR study). PLoS ONE. 2013;8(8): e70588. 10.1371/journal.pone.0070588. 23950967 10.1371/journal.pone.0070588 PMC3741197

[33] Ito T, Shimizu K, Ichida Y, et al. Usefulness of pharmacist-assisted screening and psychiatric referral program for outpatients with cancer undergoing chemotherapy. Psychooncology. 2011;20(6):647–54. 10.1002/pon.1945. 21384467 10.1002/pon.1945

[34] Ragland D, Payakachat N, Hays EB, et al. Depression and diabetes: establishing the pharmacist’s role in detecting comorbidity in pregnant women. J Am Pharm Assoc. 2010;50(2):195–9. 10.1331/JAPhA.2010.09191. 10.1331/JAPhA.2010.09191 20199962

[35] Hare SK, Kraenow K. Depression screenings: developing a model for use in a community pharmacy. J Am Pharm Assoc. 2008;48(1):4651. 10.1331/JAPhA.2008.07010. 10.1331/JAPhA.2008.07010 18192130

[36] Knight DE, Draeger RW, Heaton PC, et al. Pharmacist screening for depression among patients with diabetes in an urban primary care setting. J Am Pharm Assoc. 2008;48(4):518–21. 10.1331/JAPhA.2008.07048. 10.1331/JAPhA.2008.07048 18653429

[37] Bosmans JE, Brook OH, van Hout HPJ, et al. Cost effectiveness of a pharmacy-based coaching program to improve adherence to antidepressants. Pharmacoeconomics. 2007;25(1):25–37. 10.2165/00019053-200725010-00004. 17192116 10.2165/00019053-200725010-00004

[38] Knox ED, Dopheide JA, Wincor MZ, et al. Depression screening in a university campus pharmacy: a pilot project. J Am Pharm Assoc. 2006;46(4):502–6. 10.1331/154434506778073583. 10.1331/154434506778073583 16913394

[39] Rickles NM, Svarstad BL, Statz-Paynter JL, et al. Improving patient feedback about and outcomes with antidepressant treatment: a study in eight community pharmacies. J Am Pharm Assoc. 2006;46(1):25–32. 10.1331/154434506775268715. 10.1331/154434506775268715 16529338

[40] Crockett J, Taylor S, Grabham A, et al. Patient outcomes following an intervention involving community pharmacists in the management of depression. Aust J Rural Health. 2006;14(6):263–9. 10.1111/j.1440-1584.2006.00827.x. 17121506 10.1111/j.1440-1584.2006.00827.x

[41] Al-Saffar N, Abdulkareem A, Abdulhakeem A, et al. Depressed patients’ preferences for education about medications by pharmacists in Kuwait. Patient Educ Couns. 2008;72(1):94–101. 10.1016/j.pec.2008.01.027. 18337052 10.1016/j.pec.2008.01.027

[42] Brook OH, van Hout H, Stalman W, et al. A pharmacy-based coaching program to improve adherence to antidepressant treatment among primary care patients. Psychiatr Serv. 2005;56(4):487–9. 10.1176/appi.ps.56.4.487. 15812103 10.1176/appi.ps.56.4.487

[43] Rickles NM, Svarstad BL, Statz-Paynter JL, et al. Pharmacist telemonitoring of antidepressant use: effects on pharmacist-patient collaboration. J Am Pharm Assoc. 2005;45(3):344–53. 10.1331/1544345054003732. 10.1331/1544345054003732 15991756

[44] Brook O, van Hout H, Nieuwenhuyse H, et al. Impact of coaching by community pharmacists on drug attitude of depressive primary care patients and acceptability to patients; a randomized controlled trial. Eur Neuropsychopharmacol. 2003;13(1):1–9. 10.1016/s0924-977x(02)00074-3. 12480116 10.1016/s0924-977x(02)00074-3

[45] Bultman DC, Svarstad BL. Effects of pharmacist monitoring on patient satisfaction with antidepressant medication therapy. J Am Pharm Assoc (Wash). 2002;42(1):36–43. 10.1331/108658002763538053. 11833513 10.1331/108658002763538053

[46] Polomoff CM, Bermudez-Millan A, Buckley T, et al. Pharmacists and community health workers improve medication-related process outcomes among Cambodian Americans with depression and risk for diabetes. J Am Pharm Assoc. 2022;62(2):496-504.e1. 10.1016/j.japh.2021.10.031. 10.1016/j.japh.2021.10.031 PMC8934259 34838475

[47] Gomes NC, Abrao PHO, Fernandes MR, et al. Effectiveness of pharmaceutical care about the quality of life in patients with depression. SM J Depress Res Treat. 2015;1(1):1005. 10.36876/smjdrt.1005. 10.36876/smjdrt.1005

[48] Marques LAM, Galduróz JCF, Fernandes MR, et al. Assessment of the effectiveness of pharmacotherapy follow-up in patients treated for depression. J Manag Care Pharm. 2013;19(3):218–27. 10.18553/jmcp.2013.19.3.218. 23537456 10.18553/jmcp.2013.19.3.218 PMC10438347

[49] Canales PL, Dorson PG, Crismon ML. Outcomes assessment of clinical pharmacy services in a psychiatric inpatient setting. Am J Health Syst Pharm. 2001;58(14):1309–16. 10.1093/ajhp/58.14.1309. 11471478 10.1093/ajhp/58.14.1309

[50] Kanwal F, Pyne JM, Tavakoli-Tabasi S, et al. A randomized trial of off-site collaborative care for depression in chronic hepatitis C virus. Health Serv Res. 2018;53(4):2547–66. 10.1111/1475-6773.12758. 28891153 10.1111/1475-6773.12758 PMC6051980

[51] Greene E, McGuire M, Swift R, et al. Effect of an interdisplinary collaborative, protocol-driven intervention for depression in a minority, indigent population. J Cult Divers. 2017;24(2):46–53.

[52] Binakaj Z. Pharmaceutical care of the patients suffering from depression. J Pharm Pharmacol. 2016;4(6):253–60. 10.17265/2328-2150/2016.06.003. 10.17265/2328-2150/2016.06.003

[53] Aljumah K, Hassali MA. Impact of pharmacist intervention on adherence and measurable patient outcomes among depressed patients: a randomized controlled study. BMC Psychiatry. 2015;15(1):219. 10.1186/s12888-015-0605-8. 26376830 10.1186/s12888-015-0605-8 PMC4574071

[54] Pyne JM, Fortney JC, Curran GM, et al. Effectiveness of collaborative care for depression in human immunodeficiency virus clinics. Arch Intern Med. 2011;171(1):23–31. 10.1001/archinternmed.2010.395. 21220657 10.1001/archinternmed.2010.395

[55] Fortney JC, Pyne JM, Edlund MJ, et al. A randomized trial of telemedicine-based collaborative care for depression. J Gen Intern Med. 2007;22(8):1086–93. 10.1007/s11606-007-0201-9. 17492326 10.1007/s11606-007-0201-9 PMC2305730

[56] Capoccia KL, Boudreau DM, Blough DK, et al. Randomized trial of pharmacist interventions to improve depression care and outcomes in primary care. Am J Health Syst Pharm. 2004;61(4):364–72. 10.1093/ajhp/61.4.364. 15011764 10.1093/ajhp/61.4.364

[57] Adler DA, Bungay KM, Wilson IB, et al. The impact of a pharmacist intervention on 6-month outcomes in depressed primary care patients. Gen Hosp Psychiatry. 2004;26(3):199–209. 10.1016/j.genhosppsych.2003.08.005. 15121348 10.1016/j.genhosppsych.2003.08.005

[58] Finley PR, Rens HR, Pont JT, et al. Impact of a collaborative care model on depression in a primary care setting: a randomized controlled trial. Pharmacotherapy. 2003;23(9):1175–85. 10.1592/phco.23.10.1175.32760. 14524649 10.1592/phco.23.10.1175.32760

[59] Finley PR, Rens HR, Pont JT, et al. Impact of a collaborative pharmacy practice model on the treatment of depression in primary care. Am J Health Syst Pharm. 2002;59(16):1518–26. 10.1093/ajhp/59.16.1518. 12185826 10.1093/ajhp/59.16.1518

[60] Finley PR, Rens HR, Gess S, et al. Case management of depression by clinical pharmacists in a primary care setting. Formulary (Cleveland, Ohio). 1999;34(10):864–70.

[61] Schmidt I, Claesson CB, Westerholm B, et al. The impact of regular multidisciplinary team interventions on psychotropic prescribing in Swedish nursing homes. J Am Geriatr Soc. 1998;46(1):77–82. 10.1111/j.1532-5415.1998.tb01017.x. 9434669 10.1111/j.1532-5415.1998.tb01017.x

[62] Miller P, Newby D, Walkom E, et al. Depression screening in adults by pharmacists in the community: a systematic review. Int J Pharm Pract. 2020;28(5):428–40. 10.1111/ijpp.12661. 32776433 10.1111/ijpp.12661

[63] Al-Jumah KA, Qureshi NA. Impact of pharmacist interventions on patients’ adherence to antidepressants and patient-reported outcomes: a systematic review. Patient Prefer Adherence. 2012;6:87–100. 10.2147/PPA.S27436. 22346345 10.2147/PPA.S27436 PMC3277799

[64] Rubio-Valera M, Serrano-Blanco A, Magdalena-Belío J, et al. Effectiveness of pharmacist care in the improvement of adherence to antidepressants: a systematic review and meta-analysis. Ann Pharmacother. 2011;45(1):39–48. 10.1345/aph.1P429. 21205952 10.1345/aph.1P429

[65] Bunchuailua W, Samprasit N, Kotirum S, et al. Impact of pharmacist activities in patients with depression: a systematic review and meta-analysis of randomized controlled trials. Ann Pharmacother. 2022;56(5):556–64. 10.1177/10600280211041274. 34459265 10.1177/10600280211041274

[66] Brown JVE, Walton N, Meader N, et al. Pharmacy-based management for depression in adults. Cochrane Database Syst Rev. 2019;12(12):CD013299. 31868236 10.1002/14651858.CD013299.pub2 PMC6927244

[67] Sansone RA, Sansone LA. Antidepressant adherence: are patients taking their medications? Innov Clin Neurosci. 2012;9(5–6):41–6. 22808448 PMC3398686

[68] Hung CI, Wang SJ, Liu CY, et al. Comorbidities and factors related to discontinuation of pharmacotherapy among outpatients with major depressive disorder. Compr Psychiatry. 2011;52(4):370–7. 10.1016/j.comppsych.2010.08.005. 21683174 10.1016/j.comppsych.2010.08.005

[69] Carter BL. Evolution of clinical pharmacy in the USA and future directions for patient care. Drugs Aging. 2016;33(3):169–77. 10.1007/s40266-016-0349-2. 26895454 10.1007/s40266-016-0349-2 PMC4821736

[70] El Hajj MS, Mekkawi R, Elkaffash R, et al. Public attitudes towards community pharmacy in Arabic speaking Middle Eastern countries: a systematic review. Res Social Adm Pharm. 2021;17(8):1373–95. 33257161 10.1016/j.sapharm.2020.11.013

[71] Sallom H, Abdi A, Halboup AM, et al. Evaluation of pharmaceutical care services in the Middle East Countries: a review of studies of 2013–2020. BMC Public Health. 2023;23(1):1364. 10.1186/s12889-023-16199-1. 37461105 10.1186/s12889-023-16199-1 PMC10351150

[72] Kheir N, Zaidan M, Younes H, et al. Pharmacy education and practice in 13 Middle Eastern countries. Am J Pharm Educ. 2008;72(6):133. 10.5688/aj7206133. 19325953 10.5688/aj7206133 PMC2661169

[73] Eltorki Y, Abdallah O, Omar N, et al. Perceptions and expectations of health care providers towards clinical pharmacy services in a mental health hospital in Qatar. Asian J Psychiatr. 2019;42:62–6. 10.1016/j.ajp.2019.03.018. 30965189 10.1016/j.ajp.2019.03.018

[74] Gide DN, El-Den S, Lee YLE, et al. Community pharmacists’ acceptability of pharmacist-delivered depression screening for older adults: a qualitative study. Int J Clin Pharm. 2023;45:1144–52. 10.1007/s11096-023-01581-1. 37081167 10.1007/s11096-023-01581-1 PMC10600303

[75] Strowel C, Raynes-Greenow C, Pham L, et al. Perinatal depression screening in community pharmacy: exploring pharmacists’ roles, training and resource needs using content analysis. Int J Clin Pharm. 2023;45:1212–22. 10.1007/s11096-023-01647-0. 37792255 10.1007/s11096-023-01647-0 PMC10600310

[76] Chidambaram R. Opportunistic risk screening of depression by community pharmacists: noble intervention to mend the mind during COVID-19. Malays J Med Sci. 2022;29(4):160–4. 10.21315/mjms2022.29.4.15. 36101532 10.21315/mjms2022.29.4.15 PMC9438851

[77] National Council for Mental Wellbeing. Mental health first aid. 2023. Available from: URL: https://www.thenationalcouncil.org/our-work/mental-health-first-aid/. Accessed 07 Dec 2023.

[78] McKeirnan KC, MacCamy KL, Robinson JD, et al. Implementing mental health first aid training in a doctor of pharmacy program. Am J Pharm Educ. 2023;87(8):100006. 10.1016/j.ajpe.2023.01.001. 37597905 10.1016/j.ajpe.2023.01.001

[79] World Health Organization. World mental health day. 2023. Available from: https://www.who.int/campaigns/world-mental-health-day. Accessed 01 Dec 2023.

