# Supplementary material for: The evolving role of pharmacists in depression care: a scoping review
Source: Int J Clin Pharm. 2024 Jul 15;46(5):1044–66. doi: 10.1007/s11096-024-01759-1 (PMC11399168; doi:10.1007/s11096-024-01759-1)
Supplement: Supplementary file 1 — Supplementary file1 (DOCX 17 KB) [file 11096_2024_1759_MOESM1_ESM.docx]

**MEDLINE database**

Results:23 study

Search strategy:

Pharmacists OR Pharmacist (Title) AND Depression OR Depressive disorder (Title) AND Management OR Pharmaceutical care OR pharmacy services

TI ( pharmacists or pharmacist ) AND TI ( depression or depressive disorder ) AND AB ( management OR pharmaceutical care OR Pharmacy services )

Filters:

1- Source type: Academic journals

2- Language: English

**Scopus database**

Results: 17 study

Search strategy:

Article title: Pharmacist

AND

Article title: depression OR “depressive disorder”

AND

Article title, keywords, abstract: “pharmaceutical care” OR “Medication management OR “Pharmacy services”

Filters:

1- Document type: Limited to articles

2- Language: Limited to English

3- Keywords: No limitations

4- Source type: Limited to Journal

5- Publication stage: Final

**ProQuest database**

Results: 128 articles

Pharmacist AND TITLE(Depression) AND ABSTRACT (community pharmacy services OR medication management OR pharmaceutical care)

Filters Applied: English, scholarly journals, Articles.

**Cochrane database**

Filters: no filters

Results: 23

"pharmacist" OR "pharmacists" in Record Title AND "depression" OR "Depressive disorder" in Title Abstract Keyword AND Medication management therapy OR pharmacy service OR pharmaceutical care in Title Abstract Keyword –
